# The implications of histamine metabolism and signaling in renal function

**DOI:** 10.14814/phy2.14845

**Published:** 2021-05-01

**Authors:** Anastasia V. Sudarikova, Mikhail V. Fomin, Irina A. Yankelevich, Daria V. Ilatovskaya

**Affiliations:** ^1^ Institute of Cytology Russian Academy of Sciences St. Petersburg Russia; ^2^ Division of Nephrology Department of Medicine Medical University of South Carolina Charleston SC USA; ^3^ St. Petersburg State Chemical Pharmaceutical University St. Petersburg Russia; ^4^ Institute of Experimental Medicine St. Petersburg Russia

## Abstract

Inflammation is an essential part of the immune response; it has been found to be central to the disruption of kidney function in acute kidney injury, diabetic nephropathy, hypertension, and other renal conditions. One of the well‐known mediators of the inflammatory response is histamine. Histamine receptors are expressed throughout different tissues, including the kidney, and their inhibition has proven to be a viable strategy for the treatment of many inflammation‐associated diseases. Here, we provide an overview of the current knowledge regarding the role of histamine and its metabolism in the kidney. Establishing the importance of histamine signaling for kidney function will enable new approaches for the treatment of kidney diseases associated with inflammation.

## Graphical abstract

Inflammation is central to renal pathophysiology. Here, we provide an overview of the current knowledge regarding the role of histamine, a well‐known mediator of inflammation, and its metabolism in the kidney.

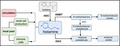

## 1 THE KIDNEY AND INFLAMMATION

Inflammation is a complex adaptive reaction aimed at eliminating external or internal pathogenic stimuli and involves both cellular and humoral factors. The process of inflammation can be triggered by various stimuli. One of the classical pathways that can activate inflammation is recognition of the pathogen‐associated molecular patterns and endogenous damage‐associated molecular patterns (DAMPs), that lead to the production of inflammatory cytokines (Janeway & Medzhitov, 2002; Zindel & Kubes, 2020). On the one hand, inflammation initiates adaptive immunity‐related processes; on the other hand, later stages of the adaptive immune response can enhance inflammation (Cronkite & Strutt, 2018). Allergen‐mediated degranulation of mast cells, which leads to a release of pro‐inflammatory cytokines and vasoactive amines (primarily histamine), is yet another trigger of inflammation (Branco et al., 2018; Thangam et al., 2018). During the inflammatory response, the blood flow to inflamed tissues is enhanced as a result of vasodilation, capillary permeability is increased, leading to recruitment of leukocytes (mainly, neutrophils) to the area of inflammation (Medzhitov, 2008). The main function of phagocytes (including neutrophils) at the site of inflammation is to ingest and destroy foreign bodies and damaged cells (Medzhitov, 2008; Rosales & Uribe‐Querol, 2017). Typically, inflammation is resolved by elimination of the pathogen that initiated it, and tissue regeneration (Headland & Norling, 2015).

Although inflammation is critically important for our body's defense mechanisms and maintenance of homeostasis, it can also lead to a pathophysiological response and tissue damage. Effector cascades employed by phagocytes to eliminate the pathogens are accompanied by the release of proteolytic enzymes and reactive oxygen species (ROS), which can be detrimental for cells and tissues (Hunter, 2012; Medzhitov, 2008). Inflammation is an essential process for a variety of pathologies, including diseases that implicate the kidney, such as diabetes, hypertension, metabolic syndrome, polycystic kidney disease (PKD), chronic kidney disease, and acute kidney injury (AKI; Furman et al., 2019; Karihaloo, 2015; Matoba et al., 2019; Rabb et al., 2016). Stressors such as a high salt diet, high blood pressure, or high glucose levels can trigger renal tissue damage and evoke an inflammatory response (Mattson, 2019). Inflammation will then cause immune cell infiltration into the kidney and production of inflammatory cytokines, which increases oxidative stress and amplifies the damage by affecting the vascular response, producing pathologies of the glomerulus and tubules, and increasing the retention of sodium and water (Mattson, 2014, 2019). Histamine, a well‐known mediator of inflammation, could represent one of the important regulators of the renal tissue function in inflammatory response (Branco et al., 2018), which will be discussed in this review.

## 2 HISTAMINE METABOLISM, AND ITS POTENTIAL RENAL SOURCES

**FIGURE 1:**
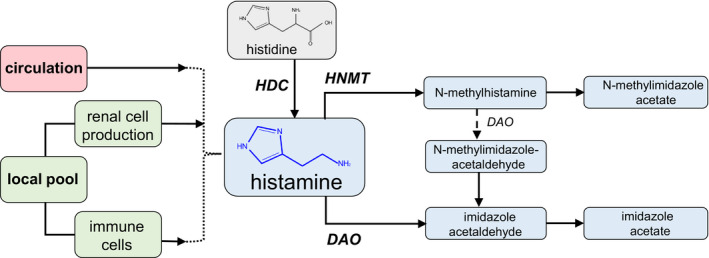
Summary of possible sources of renal histamine, and its metabolism. It is hypothesized that in the renal tissue, histamine can emerge from the circulation, or from a local pool—via intrarenal production by immune cells, such as mast cells, or renal epithelium. Histamine is formed from histidine with the help of HDC (histamine decarboxylase), and further histamine metabolites are formed by HNMT (histamine‐N‐methyltransferase) or DAO (diamine oxidase)

Histamine is a biogenic amine produced from the amino acid histidine through a removal of a carboxyl group, catalyzed by histidine decarboxylase (HDC; Huang et al., 2018). Figure 1 shows a schematic summary of histamine production and metabolism,and major enzymes involved in these processes. Many types of cells are capable of storing and releasing histamine, including mast cells, basophils, enterochromaffin‐like cells, histaminergic neurons, dendritic cells (DCs), macrophages, and even epithelial cells (Fultz et al., 2019; Huang et al., 2018; Schirmer et al., 2020; Tiligada & Ennis, 2020). When needed, histamine can be released from local stores in the tissue, or its de novo production may be triggered by a stimulus. The stored histamine is located mainly in mast cells and basophils; basophils are rare, short‐lived cells recruited to tissues upon inflammation, whereas mast cells are resident cells widely distributed throughout mucosal and connective tissues (Galli & Tsai, 2012; Hirasawa, 2019). In these cells, following its synthesis, histamine is stored in intracellular granules, in up to millimolar concentrations, until an activating stimulus triggers its release (Hirasawa, 2019; Huang et al., 2018). Histamine synthesis can also be induced in cells such as macrophages and neutrophils, from which it can be released in micromolar concentrations (Hirasawa, 2019). Histamine is metabolized via extracellular oxidative deamination catalyzed by diamine oxidase (DAO) or intracellular methylation by histamine‐N‐methyltransferase (HNMT; Comas‐Baste et al., 2020).

Multiple studies suggest that the main source of histamine in the kidney is local production. The level of the histamine metabolite N‐methylhistamine, resulting from the enzymatic activity of HNMT, is higher in the kidney as compared to most other organs (Zimmermann et al., 2011). In addition, it has been shown that histamine can be produced in isolated human glomeruli, in the absence of mast cells (Sedor & Abboud, 1984). HDC has been reported to be expressed in the human kidney, specifically in the proximal tubule cells, podocytes, distal tubules, collecting duct, and loop of Henle (according to Kidney Interactive Transcriptomics (KIT) data from Humphrey's laboratory (Wu et al., 2018)). In pregnancy, HDC is thought to have a functional role of increasing renal blood flow and recruiting immune cells to renal tissues (Morgan et al., 2006). Other local sources of renal histamine have been hypothesized, for instance, mast cells. Although the number of mast cells is constitutively low in the kidney, they can still release large amounts of histamine locally, whereas the circulatory level of histamine (10 nM) is much smaller than what was reported in the kidney (2 pmol/mg organ weight) (Grange et al., 2020; Krystel‐Whittemore et al., 2015; Zimmermann et al., 2011). Thus, the intrarenal production of histamine is likely, and has the potential to be changed during the implementation of the inflammatory response.

## 3 HISTAMINE RECEPTORS AND THEIR ASSOCIATED CASCADES

**FIGURE 2:**
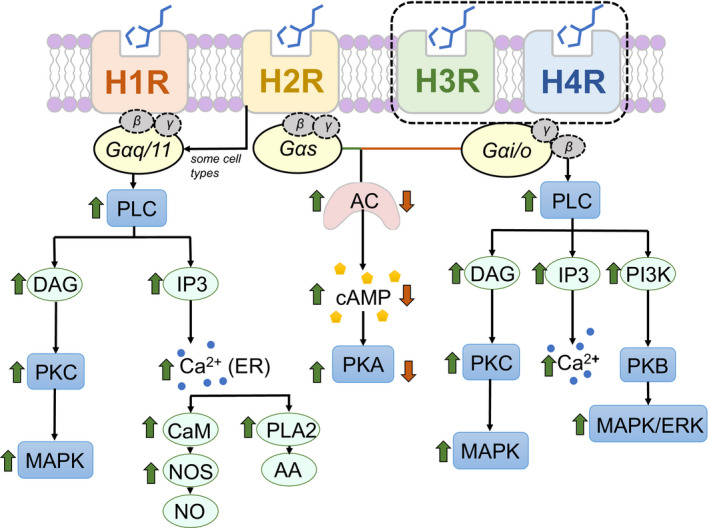
Generalized signaling pathways downstream of histamine receptors (HRs). Histamine binds to its four G‐protein‐coupled receptors: H1R, H2R, H3R, and H4R, and activates downstream signaling cascades depending on the type of G‐protein associated with each receptor. AA, arachidonic acid; AC, adenylyl cyclase; CaM, calmodulin; cAMP, 3′,5′‐cyclic adenosine monophosphate; DAG, diacylglycerol; IP3, inositol 1,4,5‐trisphosphate; MAPK, mitogen‐activated protein kinase; NOS, nitric oxide synthase; PI3K, phosphatidylinositol 3‐kinase; PKA, protein kinase A; PLA2, phospholipase A2; PLC, phospholipase C

Histamine brings about physiologic changes by binding to its four G‐protein‐oupled receptor (GPCR) subtypes: H1–H4 receptors (H1R–H4R). The basic membrane topology of each receptor includes an extracellular N terminus, an intracellular C terminus, and seven transmembrane helices interconnected by three intracellular loops and three extracellular loops (Panula et al., 2015). The receptors are constitutively active, that is, are able to adopt an active conformation independent of ligand binding (Mehta et al., 2020), but differ in their localization and downstream signaling cascades, which allows for distinctive effects in various tissues and cell types. An overview of histamine receptors and generalized downstream cascades is shown in Figure 2. Each receptor has different binding affinities for histamine, with H1R and H2R having low affinity for histamine (micromolar range), whereas H3R and H4R exhibit high affinity (5–10 nM; Mehta et al., 2020; Panula et al., 2015). The cascades downstream of each receptor are likewise unique. For instance, H1R couples to Gαq/11 proteins and activates phospholipase C‐β (PLC‐β). This catalyzes the hydrolysis of phosphatidylinositol diphosphate (PIP2) into membrane diacylglycerol (DAG) and inositol 1,4,5‐trisphosphate (IP3; Mocking et al., 2016; Panula et al., 2015). DAG activates protein kinase C (PKC), which can increase vasodilation and activate the mitogen‐activated protein kinase (MAPK) pathway. IP3 mobilizes Ca^2+^ via activation of the receptors on the endoplasmic reticulum (ER). The mobilization of Ca^2+^ can then produce such diverse effects as further release of histamine, transcription of cytokines IL‐6 and IL‐8, and activation of nuclear factor kappa (Holden et al., 2007; Mocking et al., 2016).

In response to histamine, H2R binds to a different G‐protein, Gαs, and, less often, Gαq/11, resulting in activation of adenylyl cyclase (AC) and increased cAMP levels. cAMP then stimulates protein kinase A (PKA) and induces Ca^2+^ influx to the cell. H2R has been revealed to play a role in the immune response, as inhibition of the MAPK pathway activated by H2R leads to decreased production of pro‐inflammatory tumor necrosis factors (Mocking et al., 2016; Thomas et al., 2012). Contrary to H2R, signaling through the H3R receptor is coupled to pertussis toxin‐sensitive Gαi/o proteins, and results in the inhibition of AC and subsequent reduction in formation of cAMP, and reduced PKA activity. Gβγ subunits reduce Ca^2+^ entry through voltage‐gated calcium channels and possibly activate pathways involving PLC‐IP3‐Ca^2+^ and PKC. While the basic pathway mediated by the H4R receptor is similar to H3R, this receptor has additionally been shown to be implicated in intracellular Ca^2+^ release (Hofstra et al., 2003), cell migration (Ferreira et al., 2012; Mocking et al., 2016), PI3 kinase activation, and inflammatory cytokine and chemokine regulation (Jemima et al., 2014; Mehta et al., 2020).

The HRs receptors show differential expression throughout the body. H1R is expressed in the brain, smooth muscle, endothelial cells, DCs, monocytes, and B/T cells (Panula et al., 2015; Thangam et al., 2018). H2R was shown in the brain, smooth muscle, endothelial and epithelial cells, neutrophils, monocytes, and B/T cells (Monczor et al., 2017; Panula et al., 2015). H3R is mostly expressed throughout the mature brain, but also in salivary glands, respiratory epithelium, gastric mucosa, skin, heart, and kidney at the embryonic stage of development (Grange et al., 2020; Panula et al., 2015). The H4R is most strongly expressed in the bone marrow, but its expression at the mRNA level was detected in the spleen, thymus, lung, heart, kidney, epithelial cells, and peripheral blood. The widespread pattern of HR expression is further supported by functional data in numerous immune cells, including mast cells, neutrophils, DCs, and monocytes (Kay et al., 2018; Mehta et al., 2020; Panula et al., 2015; Thangam et al., 2018).

## 4 EFFECTS OF HISTAMINE ON RENAL PHYSIOLOGY AND PATHOPHYSIOLOGY

**FIGURE 3:**
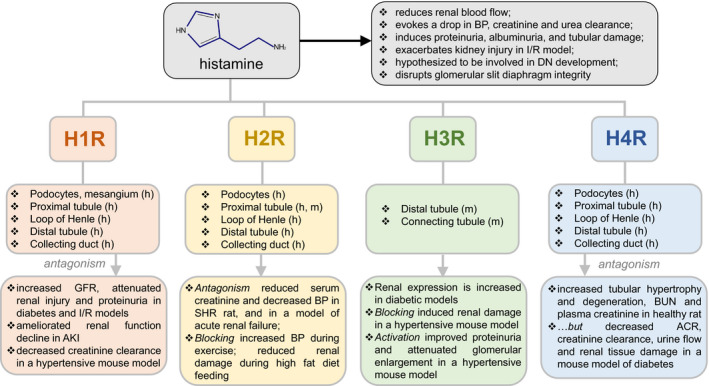
Known renal histamine receptors expression patterns and effects of their activation/antagonism in renal physiology. Shown are the known effects of histamine on renal physiology (gray), as well as the reported expression of the histamine receptors 1–4 (H1R, H2R, H3R, H4R) in various renal segments of mice (m) or humans (h) according to publicly available transcriptomic databases. The bottom row summarizes the effects of histamine receptors’ agonism or antagonism on renal physiology. ACR, albumin‐to‐creatinine ratio; AKI, acute kidney injury; BUN, blood urea nitrogen; DN, diabetic nephropathy; GFR, glomerular filtration rate; I/R, ischemia–reperfusion; BP, blood pressure

All four histamine receptors have been reported to express in the kidney, with each receptor showing a unique localization pattern. According to the KIT databases (Wu et al., 2018), in human renal tissues all four HRs can be found in the distal tubule, whereas podocytes, proximal tubules, loop of Henle, and collecting ducts express only H1R, H2R, and H4R, respectively (see Figure 3). KIT does not report H3R expression in human samples. In other species, H3R was demonstrated in the apical membrane of the principal cells of the collecting duct of the rat, whereas H4R was found at the apical membrane of the epithelial cells of the ascending loop of Henle and the proximal convoluted tubule (Grange et al., 2020; Rosa et al., 2013; Veglia et al., 2015). H1R and H2R are both expressed in the glomerular capsule, the mesangial cells, the proximal tubule, and the distal convoluted tubule (Grange et al., 2020; Veglia et al., 2015). H1R has also been found to be expressed in podocytes in humans (Grange et al., 2020). The better‐known HR is H1R, the first one to be found and originally known as “the'’ histamine receptor, now widely used for the treatment of allergies (Tiligada & Ennis, 2020). However, distinct avenues of research have been developed for each of the receptors; for instance, H2R is well known to be important for gastric acid secretion, H3R for neurotransmission, and H4R for immunomodulation. Each receptor is constitutively active, and the preferred method of treatment utilizing these receptors has been to block their activity with inverse agonists (Branco et al., 2018; Thangam et al., 2018). A list of some current pharmacology targeting histamine receptors, and specifically their known renal effects, are shown in Table 1.

**TABLE 1:** Pharmacology targeting histamine receptors and their combinations; reported are the drugs known to affect renal system function

Drug	Mode of action	IC50/EC50/pKi	Delivery	Renal effects
**H1R**				
Cetirizine	Blocker	226 nm (IC50)	Osmotic mini‐pump, 10 mg/kg/day	Decreased creatinine clearance in ANS mice model (Noguchi et al., 2020)
Desloratidine	Antagonist	51 nm (IC50), 0.87 nM (Ki)	Oral gavage, 5 mg/kg	Decreased NF‐κB, TNF‐α, IL‐1β, creatinine, BUN, reduced histopathology in renal rat I/R model (Kocaturk et al., 2020)
Levocetirizine	Antagonist	0.13 uM (IC50)	Orally, 0.5 mg/kg/day in 0.5% CMC	Increased GFR, attenuated renal hypertrophy, polyuria, proteinuria, elevation of serum creatinine and urea, kidney to body weight ratio, serum albumin, renal levels of TNF‐α and TGF‐β1, and reduced renal oxidative stress in rat diabetic model (Anbar et al., 2016)
Chlorpheniramine	Antagonist	12 nM (IC50)	pre‐treatment of immortalized podocytes, 10 μM (Veglia et al., 2016) ICV injection, 50 nmol (Jochem et al., 2016); IP injection, 20 mg/kg (Hattori et al., 2016)	Protected slit diaphragm integrity in human immortalized podocytes in response to treatment with histamine (Veglia et al., 2016); reversed leptin‐induced rise in mean arterial pressure, heart rate, and renal blood flow in rat model of hemorrhagic shock (Jochem et al., 2016); reduced blood levels of IL‐6, IL‐1β, and TNF‐α, reduced tissue levels of IL‐1β, ILβ6, and TNF‐α mRNAs, reduced levels of NGAL, serum BUN, and creatinine in mouse model of sepsis (Hattori et al., 2016)
Rupatidine	Antagonist	3.8 nM (IC50)	Oral, 3 and 6 mg/kg/day in CMC	Ameliorated diabetic nephropathy, improved histopathology, reduced fibrosis, and senescence markers in a rat model of streptozotocin‐induced diabetes (Hafez et al., 2020)
Bilastine	Antagonist	64 nM (IC50)	Oral gavage in water solution, 1–30 mg/kg	Prevented the increase in ACR, restored creatinine clearance, and preserved junctional integrity in a rat model of streptozotocin‐induced diabetes (Verta et al., 2019)
Ketotifen	Antagonist	1.3 nM (Ki)	IV injection, 1 mg/kg (Tong et al., 2016); IP injection, 1 mg/kg/day in 0.5% CMC (Reena et al., 2016)	Reduced histological injury score, BUN serum creatinine, IL‐6, and TNF‐α, downregulated expression of ICAM‐1, increased activity of SOD, in renal tissues in I/R rat model (Tong et al., 2016); attenuated HFD induced increase in body weight, kidney weight to body weight ratio, and in renal parameters (decrease in serum creatinine, increased levels in BUN, uric acid, K^+^, and microproteinuria, increase in systolic blood pressure), reversed HFD induced morphological changes in a HFD‐fed model in rats (Reena et al., 2016)
Mirtazapine	Antagonist	9.3 (pKi)	Oral gavage, 20 mg/kg/day dissolved in 2 ml saline (Sahin et al., 2019); Orally via catheter, 20 mg/kg (Tok et al., 2012)	Ameliorated glomerular and tubular histology, decreased kidney expression of caspase‐1 and fraction of TUNEL‐positive cells (apoptosis) and kidney expression of NLRP3 and IL‐1β (inflammation) in a rat model of diabetes (Sahin et al., 2019); reduced levels of lipid peroxidation and oxidative injury products in renal tissue, improved histological appearance in a rat I/R model (Tok et al., 2012)
Meclizine	Blocker	250 nM (Ki)	IP injection, 10, 30, 60, 100 mg/kg	Decreased serum creatinine, BUN, reduced tubular necrosis and kidney oxygen consumption, reduced inflammation, inhibited renal mitochondrial fragmentation in an IRI model in mice, attenuated cytochrome C release in tubular epithelial cells in vitro (Kishi et al., 2015)
Promethazine	Antagonist	0.00091 μM (IC50)	20 mg/kg in water	Mitigated renal function decline and renal tubular damage, reduced the renal lipid peroxidation, increased survival rate in mouse AKI model (Mishima et al., 2020)
**H2R**				
Famotidine	Antagonist	33 nM (IC50)	Intraperitoneal injection (20 mg/kg)	Reduced blood levels of IL‐6, IL‐1β, and TNF‐α, reduced tissue levels of IL‐1β, IL‐6, and TNF‐α mRNAs, reduced levels of NGAL, serum BUN, and creatinine in mouse model of sepsis (Hattori et al., 2016)
Ranitidine	Blocker	36–94 ng/ml (IC50)	Fed at 1.5 mg/30 g body weight	Reduced renal damage and attenuated atherosclerosis in a HFD mouse model (Liu et al., 2020)
Cimetidine	Antagonist	70 nM (Ki)	IP injection of 150 mg/kg (Estaphan et al., 2015); 0.25–1.2 mM addition to cell‐free extract (Minai‐Tehrani et al., 2011)	Decreased creatinine, BUN, K^+^, Na^+^, NO, blood pressure creatine kinase, increased GFR, urine volume, and renal glutathione (Estaphan et al., 2015); improved renal function when used in combination with L‐carnitine in a rat model of glycerol induced acute renal failure (Minai‐Tehrani et al., 2011)
**H3R**				
Carcinine	Blocker	0.29 μM (Ki)	20 mg/kg/day via osmotic mini‐pump	Decreased creatinine clearance, LVFS, increased NGAL excretion, resulted in deterioration of cardiac and renal function in ANS mice (Noguchi et al., 2020)
Immethridine	Agonist	0.85 nM (Ki)	10 mg/kg/day via osmotic mini‐pump	Increased creatinine clearance, decreased urinary albumin and NGAL, kidney NGAL mRNA expression, and urinary β2‐microglubulin, decreased perivascular fibrosis, increased kidney collagen, attenuated glomeruli and protein cast enlargement, and mesangial growth; decreased proinflammatory signal gene expression in ANS mice (Noguchi et al., 2020)
**H4R**				
JNJ39758979	Antagonist	12.5 nm (Ki)	Water solution by oral gavage, 25, 50, 100 mg/kg/day	Decreased ACR, creatinine clearance, 24 h urine volume, and urine acidification, reduced immune infiltration and fibrosis, preserved Na^+^/H^−^ exchanger 3 function in a mouse model of streptozotocin‐induced diabetes (Pini et al., 2018)
Toreforant	Antagonist	8.4 nM (Ki)	Oral gavage, 3, 10, 100 mg/kg/day in 0.5% methylcellulose	Increased renal tubular epithelial cell vacuolation, hypertrophy, degeneration, luminal dilation in the outer medulla, increased kidney weight, BUN, and serum creatinine in a 100 mg/kg/day of toreforant‐treated rat model (Ma et al., 2017)
Conessine	Antagonist	25 nM (Ki)	Oral administration, 10, 20, 50 mg/kg in water solution with combination with 7% Tween and 3% Ethanol	Increased alkaline phosphatase activity, bilirubin, urea, and creatinine concentration in liver and kidney tissues of a mouse model (Dua et al., 2013)
**H3R/H4R**				
Thioperamide	H3/H4 Antagonist	25 nM [H3] and 27 nM [H4]	Lateral cerebral ventricular injection,20 μg/10 μl	Reversed the effects of anserine on renal sympathetic nerve activity, blood pressure, and heart rate (Tanida et al., 2010)

Abbreviations: ACR, albumin‐to‐creatinine ratio; AKI, acute kidney injury; ANS, mice co‐treated with angiotensin II, nephrectomy, and salt; BUN, blood urea nitrogen; CMC, carboxymethyl cellulose; GFR, glomerular filtration rate; HFD, high‐fat diet; I/R, ischemia–reperfusion; ICAM‐1, intracellular adhesion molecule‐1; ICV, intracerebroventricular; IL‐6, IL‐1β, interleukin‐6, interleukin‐1 beta; IP, intraperitoneal; IRI, ischemia–reperfusion injury; IV, intravenous; LVFS, left ventricular fractional shortening; NF‐κB, nuclear factor kappa; NGAL, neutrophil gelatinase‐associated lipocalin; NO, nitric oxide; SOD, superoxide dismutase; TNF‐α, tumor necrosis factor‐α; TGF‐β1, transforming growth factor‐β1.

As a signaling molecule with a widespread receptor expression, histamine has effects throughout many systems (Mocking et al., 2016). In the skin, it can both amplify inflammation and participate in wound healing (Rossbach et al., 2016; Wolak et al., 2017). In the nervous system, histamine can promote the production of ROS and the loss of the mitochondrial membrane potential, and to both increase and dampen neuroinflammation (Chen et al., 2020; Kim & Song, 2017; Zhu et al., 2014). In the respiratory system, histamine can enhance the degree of fibrosis (Lucarini et al., 2016). It is the vasoactive and inflammation‐related functions of histamine that likely have the greatest relevance to renal function (Branco et al., 2018; Li et al., 2020).

Plasma histamine levels are increased in nephrotic syndrome, end‐stage renal failure, renal insufficiency, uremic pruritus, and in patients undergoing hemodialysis or peritoneal dialysis (Cao et al., 2018; Grange et al., 2020; Reszke & Szepietowski, 2018). The presence of mast cells has been correlated with loss of renal function and development of nephropathy, allograft rejection, fibrosis, and PKD (Grange et al., 2020; Holdsworth & Summers, 2008). Initial studies into the effects of histamine in the kidney were done in the 1930s with histamine challenge experiments. A low dose (0.3–0.5 mg) of histamine delivered by a subcutaneous injection resulted in an acute reduction of renal plasma flow, thought to be indicative of efferent arteriolar constriction (Bjering, 1937). A higher dose of histamine (1 mg) caused a fall in blood pressure and a drop in creatinine and urea clearance. The effect of histamine on glomerular filtration rate has been theorized to be a consequence of histamine‐mediated regulation of renal arteriolar constriction (Grange et al., 2020). Histamine has also been found to induce proteinuria, albuminuria, and degenerative tubular damage (Bjering, 1937). Furthermore, histamine was demonstrated to affect renal sympathetic nerve activity, with as low as 0.1 pM having a suppressive effect, and a 100 nM concentration having a stimulating effect (Tanida et al., 2007). In glomeruli, histamine not only induced the loss of foot processes but also disrupted cell‐to‐cell contacts and the slit diaphragm, which increased albumin excretion (Gurgen et al., 2013; Veglia et al., 2016). Histamine has also been shown to depolarize neurons that contain vasopressin, thus releasing it; vasopressin in turn stimulates water transport through the aquaporin channels (Ranieri et al., 2019). Therefore, high doses of histamine (25–500 μg i.c.v) elicit a dose‐dependent antidiuretic response with a concurrent rise in blood vasopressin in dogs (Bhargava et al., 1973).

The effect of histamine on the kidney seems to be particularly important for the development of ischemic AKI, diabetes, and hypertension. In models of AKI, administrations of DAO or HR antagonists resulted in a decrease in vascular permeability, and preservation of renal function and structural integrity (Kaneko et al., 1998). The use of cromoglicic acid, which inhibits the release of mediators by mast cells, likewise resulted in beneficial effects in this model (Tong et al., 2016). However, the use of compound 48/80, which induces histamine release, exacerbated the degree of kidney injury in the ischemic reperfusion model (Tong et al., 2016). Some studies of AKI, however, have shown a protective effect of histidine‐containing carnosine (dipeptide, beta‐alanyl‐L‐histidine), which was reversible with an H3R antagonist thioperamide (Kurata et al., 2006).

One of the leading causes of end‐stage renal disease is renal injury induced by diabetes. Lower expression or knockout of major inflammatory genes have been linked to protection against diabetes, and inhibition of inflammation has been suggested as a diabetes treatment, whereas pro‐inflammatory mediators are thought to induce diabetes progression (Hojs et al., 2016; Matoba et al., 2019; Thomas & Cherney, 2018). Histamine was also proposed to be involved in the pathogenesis of diabetic nephropathy. In earlier studies, a significant increase in the activity of HDC was reported in renal diabetic tissues (Gill et al., 1990; Markle et al., 1986). Both HDC and histamine deficiency delayed the onset of autoimmune diabetes, and decreased its incidence in the non‐obese diabetic mouse model (Alkan et al., 2015). H4R antagonism showed a protective effect in diabetic nephropathy, as indicated by reduced urine volume, pH, creatinine clearance, fibrosis, and fewer infiltrated immune cells (Pini et al., 2018; Rosa et al., 2013; Veglia et al., 2015). Treatment with H1R antagonists in a diabetic model resulted in a reduction of proteinuria and albuminuria (Anbar et al., 2016).

In hypertension, increased mean arterial pressure (MAP) causes damage to cardiac and renal tissues, which results in the release of endogenous DAMPs. In turn, DAMPs, via activation of TLRs, may induce *de novo* histamine production or histamine release by infiltrating or resident immune cells, and epithelial cells equipped with histamine synthesis machinery. Thus, antagonism of histamine receptors has been proposed for the management of cardiac damage in hypertensive diseases (Potnuri et al., 2018). H2R blocker famotidine reduced ventricular hypertrophy and improved cardiac function in a model of spontaneously hypertensive rats compared to beta‐blockers (Potnuri et al., 2018). H1R antagonist mepyramine and H2R antagonist cimetidine also inhibited a hypertensive response induced by acute restraint stress (de Almeida et al., 2015). An injection of ranitidine in embryos of the red‐footed tortoise resulted in a transient decrease in MAP and an increase in heart rate, whereas the introduction of histamine following ranitidine injection caused a transient increase in MAP and a decrease in heart rate (Crossley et al., 2013). The hypertensive effect of central injections of histamine is likely mediated by activation of the sympathetic nervous system and the release of hormones, including vasopressin, catecholamines, and angiotensin II (de Almeida et al., 2015). However, very little has been reported regarding the effects of HR antagonism or activation in renal tissues during hypertension. We can speculate about the potential effects of histamine in salt‐sensitive hypertension (SSH), a subset of hypertension affecting 30%–50% of hypertensive patients, in which a change in salt intake can produce meaningful differences in blood pressure. Salt‐sensitive (SS) subjects experience double the rate of fatal and nonfatal cardiovascular events versus non‐SS patients over the same time period (Elijovich et al., 2016; Mattson, 2014); renal inflammation is a crucial pathological feature for SSH. The latest studies suggest that a high‐salt diet produces neoantigens that lead to a release of pro‐hypertensive cytokines by the immune cells. These can trigger further cytokine release, which increases sodium retention and worsens vascular dysfunction, exacerbating the blood pressure increase (Lu & Crowley, 2018). An inflammatory response observed in SSH points to the potential importance of histamine in the orchestration of the events leading to renal damage; however, there is a gap in knowledge regarding the specific effects of histamine in this pathology.

## 5 CONCLUSION AND FUTURE DIRECTIONS

The latest research suggests that the role of histamine in the inflammatory response extends into modulation of kidney function. However, the overarching role of histamine in renal physiology, especially in renal epithelial function, has not yet been fully defined. In this review, we discussed the role of inflammation in kidney disease, as well as the importance of histamine and its receptors in renal physiology and pathology. Current literature lacks a consensus on the pattern of histamine receptors’ expression in the kidney, thus, rigorous studies with a variety of methodology and models are needed to fill this gap. Some future avenues of research may include selective in vivo blocking of each receptor, and subsequent examination of the affected pathways and related physiological functions, as well as an exploration of the exact effects of histamine in certain segments of the nephron. A determination of the precise role of histamine in renal physiology could potentially lead to novel therapies for renal inflammation‐accompanied diseases, including AKI, SSH, and diabetic nephropathy.

## DISCLOSURES

The authors have no competing interests to disclose.

## AUTHOR CONTRIBUTION

All authors participated in writing of the manuscript; the text is approved by all co‐authors. AVS and MVF contributed equally to this manuscript.

## Data Availability

The data that support the findings of this study are available from the corresponding author upon reasonable request.
